# Swiss University Students’ Risk Perception and General Anxiety during the COVID-19 Pandemic

**DOI:** 10.3390/ijerph17207433

**Published:** 2020-10-13

**Authors:** Julia Dratva, Annina Zysset, Nadine Schlatter, Agnes von Wyl, Marion Huber, Thomas Volken

**Affiliations:** 1 Department of Health, Institute of Health Sciences, Zurich University of Applied Sciences Winterthur, 8400 Winterthur, Switzerland; annina.zysset@zhaw.ch (A.Z.); nadine.wanner@gmail.com (N.S.); marion.huber@zhaw.ch (M.H.); thomas.volken@zhaw.ch (T.V.); 2 Medical Faculty, University of Basel, 4001 Basel, Switzerland; 3 Department of Psychology, Zurich University of Applied Sciences Winterthur, 8400 Winterthur, Switzerland; vonw@zhaw.ch

**Keywords:** anxiety, COVID-19, GAD-7, latent class analyses, university students, young adults

## Abstract

University students were confronted with abrupt changes to their daily lives by the COVID-19 lock-down. We investigated Generalized Anxiety Disorder Scale-7 (GAD-7) and anxiety levels, and the association between perceived impact on well-being, studies, and daily lives and anxiety levels, adjusted for gender, age, social class and affiliation. Early in the lock-down all students of the Zurich University of Applied Sciences (*N* = 12,429) were invited to a voluntary longitudinal health survey. Participation rate was 20% (*n* = 2437): 70% females, median age 25 yrs. (IQR 23–28). A total of 10% reported a deterioration of well-being compared to pre-Corona. LCA yielded three classes varying in perceived COVID-19 impact: 1 (low, *n* = 675), 2 (moderate, *n* = 1098), and 3 (strong, *n* = 656). Adjusted proportion of moderate to severe anxiety by class were 45% (95% CI: 28.0–62.0), 15.5% (95% CI: 13.1–17.9), and 5.1% (95% CI: 4.7–5.6), respectively. Multivariate regression analyses yielded an OR for moderate to severe anxiety of 3.88 (95% CI: 2.5–6.0, class 2) and 22.43 (95% CI: 14.5–34.6, class 3) compared to class-1. The investigated association implies that containment measures have a selective effect on anxiety in students. The diversity of students’ perception and associated anxiety should be monitored and considered in future response to pandemics.

## 1. Introduction

Public health measures implemented in reaction to COVID-19 had an enormous impact on daily lives of all citizens. Similar to other countries, and in view of dramatic developments in Italy [1], a neighboring country, Switzerland introduced containment policies including closure of schools and universities (17 March 2020), restriction of gatherings to maximum of five persons together with a general recommendation to stay at home whenever possible (21 March 2020), next to preparing the health services for a high number of infected in need of intensive medical care. University students are not considered a risk group for COVID-19; however, the threatening news can potentially cause stress, fear [2], concern for family members, and drastic changes in daily routines, social contacts, and finances [3]. The United Nations Educational, Scientific and Cultural Organization (UNESCO) estimates that 60% of all students globally are affected by school closures [4], and numerous experts anticipate an adverse impact on mental health due to the pandemic and its containment measures [5,6,7,8,9]. Mental health problems and disorders are a serious public health issue in students with a fifth of students affected worldwide [10,11]. General population studies find current prevalence rates of 1.5% to 3.0%, lifetime rates of 4.0–7.0% [12,13,14], and substantial burden and impairment [15]. 

Despite recent large-scale epidemics and pandemics, such as SARS, H1N1 avian flu or Ebola, very little research has been performed on the effect of these acute and personally uncontrollable stressors in students’ mental health or the role of universities’ in alleviating the stress [16,17]. A recent rapid review on behaviors and mental health outcomes in pandemics and general populations indicates that, among the outcomes investigated in the past, anxiety and worry can have a significant impact on the daily life and work [18,19]. The prevalence and severity of anxiety and worry, feeling of panic, depression and emotional disturbance, was initially high, decreasing over time [19].

First evidence on adverse impact by COVID-19 on students’ mental health comes from China. Cao et al. studied students at Changzhi medical college and Zhou et al. researched a Chinese adolescent population [20,21]. A study from Saudi Arabia investigated medical students and found anxiety to be associated with increased hygiene and social avoidance behavior [22]. A longitudinal study in Switzerland investigated emotional distress and concluded an increase and association with prior resilience and coping strategies [23].

The “**HE**alth in **S**tudents study during the **C**orona pandemic” (HES-C) aims to (1) evaluate the mental situation of students during the epidemic, (2) investigate changes in health behavior and associated factors, as well as (3) study student’s perception of the pandemic and related measures and their impact on students’ lives. This paper investigates the impact on student’s daily lives and how this relates to their anxiety levels.

## 2. Materials and Methods

Students from all faculties of the Zurich University of Applied Sciences (ZHAW) (*N* = 12,431), a university of applied sciences in the German speaking part of Switzerland, received a non-personalized email inviting them to participate in the study, providing study information, and a link to the survey and the study homepage. The study was approved by both the local cantonal ethics committee (BASEC-Nr. Req-2020–00326) and the ZHAW data protection officer. The baseline survey ran from 3 to 14 April 2020. The net participation rate was 20%. Participation rates by department and respective to department student number differed: Applied Linguistics 23%, Applied Psychology 30%, Architecture, Design and Civil Engineering 13%, Health Profession 35%, Life Science and Facility Management 21%, School of Engineering 17%, School of management and Law 11%, and Social Work 31%. 

The questionnaire contained internationally accepted standardized instruments and questions to assess subjective wellbeing, health status, mental health and health behavior, as well as a set of questions on COVID-19 pandemic, partly specifically developed for this target group and their context and partly adapted from a Swiss survey addressing the general population [24] (see online supplement Table S1: Overview of HES-C topics and instruments). The questionnaire was developed by the authors representing researchers, lecturers and students. Five students of different faculties piloted the questionnaire reporting any technical and content issues. Our target outcome, anxiety, was measured with the General Anxiety Disorder-Scale-7 (GAD-7; Spitzer, Kroenke, Williams and Löwe, 2006). The GAD-7 is a self-assessment questionnaire that measures the level of perceived anxiety in the last two weeks. The questionnaire is comprised of 7 items to be rated on a 4-point Likert scale (from “not at all” to “nearly every day”). The resulting sum score ranges from 0 to 21, with lower values indicating lower level of anxiety, and is categorized into four severity levels of anxiety: minimal (0–4), mild (5–9), moderate (10–14) and severe (15–21). 

Students were asked about the effects of COVID-19 pandemic and the public health measures on their subjective wellbeing and their students’ and everyday life. They answered to questions (Q1) and (Q2) “How are you doing at the moment/were you doing before the pandemic?” on a 5-point Likert scale (1 = very poor to 5 = very good), and were asked to agree or not agree to provided statements on a 5-point Likert scale (1 = strongly disagree, 5 = strongly agree, “not relevant”). The statements regarding students life were: (Q3) My timetable and thus my daily structure has changed a lot; (Q4) My weekly workload has reduced; (Q5) My weekly workload has increased; (Q6) I miss the social contacts with my fellow students; (Q7) The lecturers are available as contact persons for the students; (Q8) The higher proportion of self-study allows me more freedom; (Q9) The higher proportion of self-study is a huge challenge for me; (Q10) I am well informed about the consequences of the ZHAW’s decisions concerning my semester examinations; (Q11) I am worried about my semester degree; (Q12) I can carry out my planned internships (during my studies or during the summer holidays). Statements on everyday life were taken from a survey by the national broadcasting service [24]: (Q13) I experience more tense and conflict; (Q14) I am lonely; (Q15) I experience a stronger neighborhood; (Q16). I am bored; (Q17) I feel locked up; (Q18) I enjoy time with my family/partner; (Q19) Childcare is challenging; (Q20) No specific impact.

We also assessed the concerns students had about themselves or their family (parents, siblings, grandparents, own child/child of partner, other relative). They could answer the question “Are you concerned about your own health in the context of the pandemic?” with either “I have no concerns” (=1), “…some concerns” (=2) and “…big concerns” (=3). For family members financial concerns were addressed in the same manner, omitting the question on “child”.

Sociodemographic variables collected were age (year of birth), gender, nationality, university departmental affiliation, subject of study, pursued degree (BSc., MSc.), part-time vs. full-time study and living situation, currently and before the pandemic. Subjective parental social status at student age 16 years. (McArthur Scale, [25]) and place of birth was assessed. 

### Statistical Analyses

In order to capture variations in students’ subjective wellbeing and perceptions of everyday life during the COVID-19 lockdown, we employed a latent class analysis (LCA) of the multinomial family with logit link. Classes were inferred from all 20 available indicators on subjective wellbeing and students’ perceptions of everyday life. Indicators leading to boundary parameter estimates, i.e., probabilities very close to zero or one (logit +/− 15.0), were dropped from the pool of indicators as they cause numerical problems in estimation algorithms [26], may indicate identification problems or convergence to a local likelihood maximum [27]. In the final LCA, the following indicators were included: (Q1), (Q8), (Q9), (Q11), (Q13), (Q14), (Q17), (Q18), and (Q20). A total of 5 LCA models involving one to five latent classes were estimated using the maximum likelihood method. Based on the Bayesian information criteria (BIC), the model with three latent classes was selected. 

In a first step, ordinary least-square (OLS) regression with robust standard errors were used to estimate unadjusted and adjusted models for anxiety. All models included the LCA derived classes. Moreover, adjusted models included gender, age (centered at the mean value), parental social status (centered at the mean value), foreign nationality (reference: Swiss nationality), and university department affiliation (reference: department of health professions). 

In a second step, we used binary logistic regressions with robust standard errors to estimate unadjusted and adjusted models for anxiety at pre-defined, clinically relevant cut-off points for severe level of anxiety (GAD-7 ≥ 15 and moderate to severe level of anxiety (GAD-7 ≥ 10) [28]. 

We used Stata Version 15.1 (StataCorp, College Station, TX, USA) for all statistical analyses. Statistical significance was established at *p* < 0.05. 

## 3. Results

The study sample contains students from all faculties (*N* = 2429), students from the school of health professions and social work were slightly overrepresented (Table 1). A total of 70% were female students and the median age was 25 years (IQR 23–28); 12% reported feeling “poor” to “very poor” (current wellbeing); 10% of the students (*n* = 196), who had felt “very good”, “good” or “fair” before the pandemic now reported feeling “very poor” or “poor”, while among those 57 students who had felt poorly before, about two-thirds indicated an improved wellbeing (“fair”–“very good”, *n* = 39). Students who answered the question on health concerns regarding COVID-19 (*N* = 2080) voiced little concern for themselves (some concern 41.0%, big concern 2.7%), while major concern for relatives was frequent (some concern: parent (p) 53.1%, grandparents (gp) 49.6%; major concern: p 23.8%, gp 41.1%).

Table 2 shows the results of the GAD-7 scale. Of the 2429 university students, 2223 students filled out the GAD-7. Most students indicated minimal (38.6%) and mild symptoms of anxiety (38.6%), while a about a quarter fell into the severity categories of moderate (10–14) and severe anxiety (15–20) [28]. 

### 3.1. Latent Class Analysis: Wellbeing and Perception of Everyday Life during Lockdown

Latent class marginal probabilities for classes 1, 2, and 3 were 0.285 (95% CI: 0.255–0.318), 0.441 (95% CI: 0.403–0.479), and 0.274 (95% CI: 0.240–0.311). 

**Figure 1:**
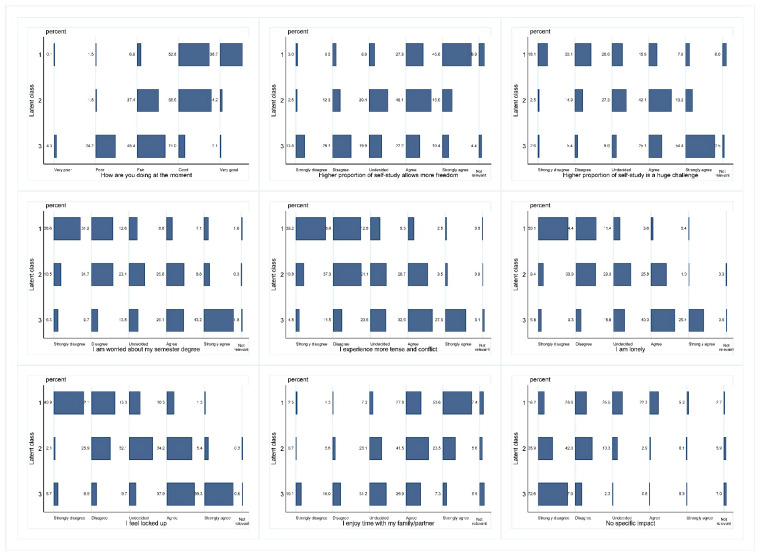
Latent class patterns. Legend: *N* = 2′429; Latent class 1 = low; 2 = moderate, 3 = high impact on wellbeing and daily life, based on latent class analysis.

The three latent classes represent clearly distinct profiles (Figure 1). Class 1 comprises students who were generally feeling good or very good, are better able to cope with the challenges induced by self-study, are less worried about their semester degree, perceive less changes in the intensity of conflict, do not feel lonely or locked up, and are better able to enjoy time with their family or partner. Moreover, roughly 30% of the students in class 1 agreed that the lockdown did not have an impact on their daily lives. 

Class 3 comprises students who generally indicate lower degrees of wellbeing, perceive self-study more challenging, are more worried about their semester degree, experience more tense conflicts, feel more lonely and locked up, are more ambivalent about whether they enjoyed time with their family or partner, and only 8.1% of the students in class 3 indicated that the lockdown did not have an impact on their everyday life. In many respects, class 2 is more similar to class 1; however, students are more often undecided, with large proportions of students in the adjacent categories (disagree or agree).

### 3.2. Anxiety

**Figure 2:**
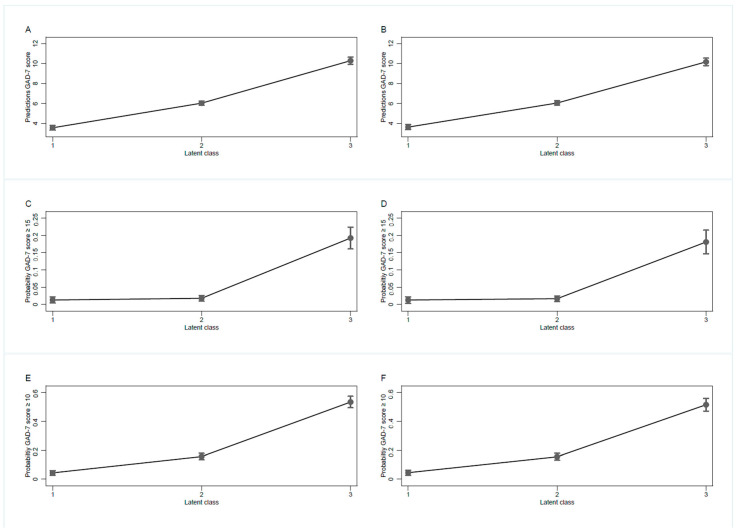
GAD-7 Predicted Marginals. Legend: *N* = 2′223; Latent class 1 = low; 2 = moderate, 3 = strong impact on wellbeing and daily life, based on latent class analysis; GAD 7: General anxiety disorder scale; unadjusted estimates (prediction and probability): A, C, E, estimates adjusted for age, sex, social status and affiliation: B, D, F. A and B estimated from OLS regression models with robust standard errors. C, D, E, and F estimated from logistic regressions with robust standard errors.

Students’ wellbeing and perceptions of everyday life were associated with anxiety in both OLS models. Compared with students belonging to class 1, students in classes 2 and 3 had substantially higher anxiety scores (Figure 2). 

Unadjusted and adjusted effects of class membership were of similar magnitude. In the adjusted OLS model, students anxiety score was 2.4 points (95% CI 2.1–2.8; *p* = 0.000) higher in class 2 and 6.5 points higher (95% CI 6.1–7.0; *p* = 0.000) in class 3 as compared to students in class 1 and the respective predicted anxiety scores, with covariates at their mean value, were 3.6 for class 1 (95% CI: 3.4–3.9; *p* = 0.000), 6.1 for class 2 (95% CI 5.8–6.3; *p* = 0.000), and 10.2 for class 3 (95% CI: 9.8–10.6; *p* = 0.000) (Table 2). 

Regression analyses (Table 3) show that men, compared to women, had somewhat lower anxiety scores (−1.3; 95% CI: −1.8–−0.9; *p* = 0.000). Furthermore, anxiety scores differed slightly between faculties. Age, parents’ social status, and nationality were not associated with anxiety.

#### 3.2.1. Severe Anxiety

Adjusting for gender, age, social class and affiliation had no substantial effect on the probability of belonging to the group with severe anxiety (Table 2 and Table 3). In the adjusted logistic regression model, students in class 3 had substantially higher odds of severe anxiety levels (OR = 16.7; 95% CI: 7.8–35.5; *p* = 0.000) as compared to students in class 1. However, the odds of severe anxiety did not significantly differ between class 1 and class 2 students (OR = 1.3; 95% CI: 0.6–3.0; *p* = 0.554). Men as compared to women had substantially smaller odds of reporting severe anxiety (OR = 0.5; 95% CI: 0.3–0.9; *p* = 0.013), while foreign nationals as compared to Swiss nationals had higher odds (OR = 2.3; 95% CI: 1.2–4.3; *p* = 0.012). Similarly, students of applied linguistics (OR = 2.4; 95% CI: 1.1–4.9; *p* = 0.020) and life sciences and facility management (2.4; 95% CI: 1.2–4.9; *p* = 0.013) had higher odds as compared to students of health professions. No statistically significant differences were found between the latter and students of the remaining faculties, nor for age and parents’ social status.

#### 3.2.2. Moderate to Severe Anxiety

Again, adjustment yielded little change to the results. The adjusted analyses resulted in more than half of the latent class 3 students (53.3%) reporting moderate to severe anxiety as compared to 4% in class 1 or 16% in class 2 (Table 2). 

In the adjusted logistic regression model (Table 3), students in classes 2 and 3 had substantially higher odds of moderate to severe anxiety levels (OR = 3.9; 95% CI: 2.5–6.0; *p* = 0.000) and (OR = 22.4; 95% CI: 14.5–34.6; *p* = 0.000) as compared to students who were little affected by the lockdown (class 1). Men as compared to women had smaller odds of belonging to the moderate to severe anxiety level group (OR = 0.5; 95% CI: 0.4–0.7; *p* = 0.000), as did students with higher social status parents (OR = 0.91; 95% CI: 0.85–0.99; *p* = 0.026) while older students (OR = 1.02; 95% CI: 1.00–1.04; *p* = 0.044) had a slightly higher odds. Students of applied linguistics (OR = 1.7; 95% CI: 1.1–2.7; *p* = 0.027), life sciences and facility management (1.8; 95% CI: 1.2–2.7; *p* = 0.006), and management and law (OR = 1.6; 95% CI: 1.1–2.3; *p* = 0.020) had higher odds of moderate to high anxiety levels as compared to students of health professions. 

## 4. Discussion

In this cross-sectional survey, capturing the early phase of the lock-down in Switzerland, a quarter of students showed moderate to severe generalized anxiety. Latent class analyses identified three distinct groups of students, who differ significantly in the impact the COVID-19 pandemic had on their well-being and daily lives. In fact, the high overall prevalence of severe anxiety is driven by students perceiving strong impact by containment measures. Students who felt strongly affected by the measures not only yielded a more than 20-fold adjusted odds of severe anxiety but also had 10- to 14-fold higher within group prevalence of severe anxiety. 

The latent class analysis supported the assumption that the subjective perception of the impact differs across student populations. Three classes proved to capture underlying clusters best, resulting in classes of little, moderate, and strong perceived effect of the COVID-19 pandemic on wellbeing and everyday life. The latent classes differentiate those students who appreciate the higher freedom that comes with self-study from those who perceive self-study as a “huge challenge” and are worried about their degrees. The previous literature indicates that abrupt changes and disruption of academic routine can cause considerable distress [9]. Next to university life, daily life was impacted both positively and negatively with increased stress at home, but also enjoying time with one’s family. The feeling of loneliness and being locked up was also mentioned far more often by students of the latent class 3.

The most widely applied measure of anxiety used both in clinical practice and research for screening, diagnosis, and assessment of the severity of anxiety disorders is GAD-7 [29]. The overall mean anxiety score found in our baseline survey was 6.5 and lies above the mean anxiety score in most of the more recent students’ health studies. Anwer et al. report a GAD-7 score of 5.3 in a Saudi Arabian college and university sample [30], and Al-Rabiaah a GAD-7 score of 2.7 (SD 3.1) in Saudi Arabian medical students [22]. Lu et al., in a Chinese study, found a mean GAD-7 score of 4.8 (SD 3.8) [31]. 

Our analyses indicate that COVID-19-specific factors are clearly related to anxiety in students and the prevalence of anxiety. Half of the latent class 3, i.e., students who perceived a strong effect on well-being and everyday life, scored above 10, the cut-off for moderate anxiety, while students, moderately (latent class 2) or little affected (latent class 1) only 16% and 4.5%, respectively, showed corresponding scores. Additional factors associated with high anxiety scores were female gender, higher age, lower perceived parental social status, and faculty affiliation. Most studies identify a higher reporting of anxiety by females as compared to males, and also report an earlier manifestation, higher comorbidity, and a worse outcome [32], as well as increasing prevalence with age [12,13]. In our study, however, adjusting for gender did not change the overall probability of experiencing moderate–severe or severe anxiety levels, nor was gender a significant factor in the logistic regression models on anxiety levels.

Differences in reported anxiety by affiliation have been reported but are less consistent in the literature. In our data, students from the department of health professions showed significantly less anxiety compared to students of management and law, life sciences or linguistics. Health profession students were also significantly less prevalent in latent class 3 as compared to other faculties. Most likely, professional knowledge and experience regarding infection risk and hygiene measures play a role. A pre-COVID university study found differences by faculties, with lowest anxiety in health professions and engineering students [33], while a global study in medical students observed higher general anxiety prevalence in medical students compared to same-age peers [34]. 

Thus far, a paucity of data in this specific age group collected during the pandemic exist. The study by Elmer et al. in a about 200 students supports our hypothesis of an adverse impact of COVID-19 on anxiety levels, but anxiety was not their main outcome, and partially differs with respect to “concern”. The authors found a high prevalence of concern for the participants’ own and family members’ health, while in our study sample, students reported little concern for their own health, but a third and a quarter reported big concern for grandparents and parents, respectively. Three Chinese studies investigated mental health in college students. Cao et al. report a prevalence of 0.9% severe, 2.7% moderate, and 21.3% mild anxiety in 7143 medical college students [20], very similar prevalence was found by Chang et al., with 0.7%, 2.71%, and 23.19%, respectively [35]—only an abstract paper in Chinese. The third conducted a longitudinal survey in 66 students and found that the COVID-19 death count had an indirect effect on negative emotions, anxiety and stress mediated through sleep quality [36]. Cultural differences in reporting emotions, anxiety and the regional discourse on COVID-19 and death toll most likely play a role in the magnitude of anxiety found in the different studies. Overall, COVID-19 studies in students are in line with our findings. Further studies on mental health in similar age groups that did not assess anxiety levels, point to the same direction of a negative impact of COVID-19 on other mental health outcomes [23,37]. The body of evidence, albeit still small, indicates the need to enhance mental health services in universities when such acute stressors occur. Universities can offer access to low-threshold support and therapy and thus reduce short- and long-term impact of crises such as pandemics.

As for associated factors Cao et al. report that living alone, coming from a rural area, financial insecurity of family was associated significantly with higher anxiety but not gender or age [20]. An Italian survey on psychological distress in the general population also yields an increased risk for anxiety when living alone and confirms our result on female gender [38]. While we investigated the personal impact on well-being and daily life as well as health concern, other studies asked students if a relative or acquaintance was infected with COVID-19 [20] or was subjected to quarantine [38]. Both found a positive association with these factors and the level of anxiety, underlining that personal experience during the COVID-19 pandemic plays an important role with regard to the COVID-19 impact on mental health. 

A limitation of our study is that we cannot infer a causal association between the perceived impact of the public health measures and reported anxiety. However, we do find higher levels of anxiety than pre-COVID-19 studies did. Another limitation is the non-random sampling method (open cohort design) method and the participation rate of 20% that may have led to some selection bias. However, we find the expected associations with known explanatory characteristics [20,33,39] and students were not informed on the health topics to be investigated. Further, by introducing latent classes, we corroborate not only the hypothesis of a differential perception of the pandemic, but also limit selection bias affecting the observed association between the perception of COVID-19 measures and anxiety levels. 

## 5. Conclusions

The data indicate that some students deal better with the dramatic changes they experienced, such as students in health professions, younger and more affluent students. Others fare less well, and with respect to future measures public health and educational institutions should address their needs. The pandemic may reverberate in higher education much longer after the outbreak has been finally controlled and a one-size-fits-all response will not suffice; thus, the authors suggest a close monitoring of health and academic outcomes of university students, and corresponding actions if needed. 

## Figures and Tables

**Table 1:** Participants’ characteristics by latent classes.

Characteristics	Total (*N* = 2429)	Latent Class 1 ^1^ (*n* = 675)	Class 2 ^1^ (*n* = 1098)	Class 3 ^1^ (*n* = 656)
	M (SD)/%	M (SD)/%	M (SD)/%	M (SD)/%
Age (years) **	26.4 (5.6)	26.9 (5.9)	26.0 (5.2)	26.6 (5.9)
Gender				
Male	30.0	32.1	28.8	29.9
Female	69.9	67.6	70.9	69.4
Others	0.4	0.3	0.3	0.8
Affiliation (Department) ***				
Applied Linguistics	7.9	6.4	8.3	9.0
Applied Psychology	7.0	8.0	6.3	7.4
Architecture, Design and Civil Engineering	2.4	3.0	2.5	1.7
Health Professions	24.5	28.4	26.4	17.6
Life Sciences and Facility Management	12.4	12.1	12.7	12.4
School of Engineering	14.7	15.7	13.0	16.7
School of Management and Law	20.9	16.3	21.1	25.6
Social Work	9.8	10.1	9.8	9.5
Bsc./Msc. Level **				
Bachelor students	87.2	84.4	86.9	90.3
Master students	12.8	15.6	13.1	9.5
Study mode				
Part-time studies	34.1	35.7	33.0	35.1
Full-time studies	65.4	64.3	67.0	64.9
Nationality (*n* = 2017)				
Swiss	73.8	76.5	72.7	72.8
Swiss dual citizenship	17.9	15.0	19.0	19.0
Foreign nationality	8.3	8.5	8.3	8.2
Subjective Parental Social Status at age 162 ***	5.64 (1.64)	5.74 (1.65)	5.73 (1.56)	5.37 (1.78)

^1^ Latent class 1 = low; 2 = moderate, 3 = high impact on wellbeing and daily life, based on latent class analysis; 2 measured by McArthur Scale [25]. *p*-value *** <0.001, ** <0.01.

**Table 2:** Anxiety sum score and severity levels, unadjusted and adjusted proportions, by latent class (*n* = 2179).

	Total (*n* = 2223)	Latent Class 1 ^1^ (*n* = 615)		Class 2 ^1^ (*n* = 1004)		Class 3 ^1^ (*n* = 604)	
GAD-7 sum score ^2^ M (SD)	6.5 (4.4)	3.6 (3.1)		6.0 (3.5)		10.3 (4.5)	
GAD-7 categories (in %)		unadj.	adj. (95% CI) ^3^	unadj.	adj. (95% CI) ^3^	unadj.	adj. (95% CI) ^3^
Minimal (0–4)	38.6	69.1		38.4		7.8	
Mild (5–9)	38.6	26.5		45.9		38.9	
Moderate (10–14)	16.4	3.1		13.8		34.1	
Severe (15–21)	6.4	1.3	1.3 (0.4–2.2)	1.8	1.7 (0.9–2.5)	19.2	18.1 (14.6–21.5)
Moderate to severe (>10)		4.4	4.5 (2.8–6.2)	15.6	15.5 (13.1–17.9)	53.3	51.4 (47.0–55.9)

^1^ Latent class 1 = low; 2 = moderate, 3 = high impact on wellbeing and daily life, based on latent class analysis; ^2^ GAD-7: General anxiety disorder scale; ^3^ adjusted for age, sex, social status and affiliation.

**Table 3:** Association between latent classes and General Anxiety Disorder scale (GAD-7).

	Linear Regression Model		Logistic Regression Model			
	GAD-7 Score ^2^ (*n* = 1983)		GAD-7 ^2^ ≥ 15 (*n* = 1976)		GAD-7 ^2^ ≥ 10 (*n* = 1983)	
Variable	*b*	95% CI	OR	95% CI	OR	95% CI
Latent class (ref = 1) ^1^						
Class 2 ^1^	2.41 ***	2.07–2.75	1.29	0.55–3.04	3.88 ***	2.51–5.99
Class 3 ^1^	6.53 ***	6.07–7.00	16.69 ***	7.85–35.48	22.43 ***	14.53–34.63
Gender (ref = female)						
Male	−1.34 ***	−1.75-(−0.93)	0.52	0.31–0.87	0.54 ***	0.39–0.74
Other	−1.45	−3.80–0.90	1.00		0.28	0.02–3.15
Age (years)	0.02	−0.01–0.05	1.02	0.98–1.06	1.02 *	1.00–1.04
Parent’s social status ^3^	−0.10	−0.21–0.00	1.07	0.94–1.21	0.91 *	0.85–0.99
Nationality (ref = Swiss)						
Swiss dual nationality	−0.13	−0.55–0.30	1.15	0.70–1.91	0.81	0.59–1.13
Foreign nationality	0.47	−0.16–1.11	2.27 *	1.20–4.27	1.22	0.80–1.85
Faculty (ref = health professions)						
Applied Linguistics	1.26 ***	0.63–1.90	2.36 *	1.14–4.86	1.68 *	1.06–2.65
Applied Psychology	0.31	−0.39–1.00	1.41	0.57–3.48	1.00	0.57–1.75
Architecture, Design and Civil Engineering	0.12	−0.70–0.95	1.89	0.44–8.18	0.86	0.34–2.19
Life Sciences and Facility Management	1.26 ***	0.71–1.81	2.42 *	1.20–4.88	1.79 **	1.18–2.73
School of Engineering	0.44	−0.16–1.05	1.47	0.66–3.29	1.50	0.94–2.40
School of Management and Law	0.76 **	0.25–1.28	1.76	0.93–3.34	1.56 *	1.07–2.27
Social Work	0.55	−0.09–1.20	1.69	0.78–3.70	1.18	0.73–1.89
Constant	3.45 ***	3.08–3.83	0.01 ***	0.00–0.02	0.04 ***	0.03–00.7

*** *p* < 0.001, ** *p* < 0.01, * *p* < 0.05. ^1^ Latent class 1 = low; 2 = moderate, 3 = high effect on wellbeing and daily life, based on latent class analysis; ^2^ GAD-7: General anxiety disorder scale; ^3^ defined by Subjective social status of parents [25] (SSS), Model *n* < GAD-7 data/*n* = 2′223) sample due to missing covariates.

## Supplementary Materials

The following are available online at https://www.mdpi.com/1660-4601/17/20/7433/s1, Table S1: Overview of topics and instruments HES-C Questionnaire.
