# Supplementary material for: Swiss University Students’ Risk Perception and General Anxiety during the COVID-19 Pandemic
Source: Int J Environ Res Public Health. 2020 Oct 13;17(20):7433. doi: 10.3390/ijerph17207433 (PMC7599649; doi:10.3390/ijerph17207433)
Supplement: Supplementary file 1 [file ijerph-17-07433-s001.pdf]

Swiss students' risk perception and general anxiety in the COVID-19 pandemic

Julia Dratva [1,2], Annina Zysset[1], Nadine Schlatter[1], Agnes von Wyl[3], Marion Huber[1], Thomas Volken[1]

[1] Zurich University of Applied Sciences Winterthur, Departement of Health, Institute of Health Sciences

[2] University of Basel, Medical Faculty

[3]Zurich University of Applied Sciences Winterthur, Departement of Psychology

Supplemental Table. 1: Overview of topics and instruments HES-C Questionnaire

Topic	Indicator	Instrument
Sociodemographic	Age	Year of birth
	Gender	Male, Female, Other
	Study	Department, Level of study, Study situation
	Residence	Residence before pandemic, currently
	Children	Number of children in household
	Migration background	Nationality; country of birth father; country of birth mother (SGB, 2017)
	Social status	Subjective social status of parents (SSS; Hoebel et al., 2015)
COVID	COVID symptoms	COVID symptoms, tested for COVID (last 4 weeks), test result
	Everyday life in the Corona crisis	Effects of everyday study work, Effects of everyday life at home (SRG, 2020)
	Public health recommendations	Grade of compliance Reasons for going outside the house (SRG, 2020)
	Information behaviour	Information sources, Frequency of utilization, Change in utilization
	Confidence	Institutional confidence (competence, the openness of communication, measures)
	Concern about family	Concerns about health and economic situation of family members (parents, grand-parents, siblings, child, other)
Mental health	Stress	Perceived Stress Scale (PSS-10; Klein et al., 2016)
	Anxiety Disorder	Generalized Anxiety Disorder 7 Questionnaire (GAD-7; Spitzer et al., 2006)
	Self-efficacy	Short Scale for Measuring General Self-efficacy Beliefs (ASKU; Beierlein et al., 2012)
	Resilience	Brief Resilient Coping Scale (BRCS; Sinclair & Wallston, 2004)
	Depression	Patient Health Questionnaire (PHQ-9; Kroenke et al., 2001)
Health	State of health	At the moment and before the pandemic
	BMI	Self-reported weight (kg) and size (cm) (SGB, 2017)
	Pain	Frequency of neck or back pain, headaches, abdominal pain, stomach ache (last 30 days, adapted HBSC 2019)
	Chronic diseases	Available / Not available (SGB; 2017)
	State of health	Current health condition (adapted; SGB, 2017)
	Concerns	Concerns about own health (SRG, 2020)
Health behaviour	Physical activity	Intensive and moderate physical activities (adapted, IPAQ SF; Craig et al., 2003; adapted MenuCH, 2015), Seated activity (adapted SGB, 2017; IPAQ SF; Craig et al., 2003)
	Nutrition	Daily meals, Snacking, Drinking (adapted menuCH, 2015)
	Alcohol consummation	Frequency, Quantity, Binge (adapted, ESPAD, 2009), Change under COVID-19

	Tobacco consummation	Smoking status (SGB, 2017), Average consumption of tobacco products (adapted; SGB, 2017), Change under COVID-19
	Marijuana consummation	Average consumption of marijuana (adapted; ESPAD, 2017), Change under COVID-19
Resources/ Burdens	Social support	Oslo Social Support Scale (Oslo-3; Kocalevent et al., 2018)
	Social capital/Anomy	Social Trust Scale (SST; Breyer, 2015)
Economic situation	Employment	Employment, workload (%) (adapted BFS, 2016), Change of workload (%) due to COVID-19

The full questionnaire can be requested from the author.
